# Diagnostic accuracy and confounders of vagus nerve ultrasound in amyotrophic lateral sclerosis—a single-center case series and pooled individual patient data meta-analysis

**DOI:** 10.1007/s00415-024-12601-z

**Published:** 2024-07-31

**Authors:** Katharina J. Müller, Moritz L. Schmidbauer, Sonja Schönecker, Katharina Kamm, Johann O. Pelz, Korbinian Holzapfel, Marianna Papadopoulou, Eleni Bakola, Georgios Tsivgoulis, Markus Naumann, Andreas Hermann, Uwe Walter, Konstantinos Dimitriadis, Peter Reilich, Florian Schöberl

**Affiliations:** 1 Department of Neurology With Friedrich Baur Institute, LMU University Hospital, LMU Munich, Munich, Germany; 2 Department of Neurology, Leipzig University Hospital, Leipzig, Germany; 3 Department of Neurology and Clinical Neurophysiology, University of Augsburg, Augsburg, Germany; 4 Department of Physiotherapy, Laboratory of Neuromuscular and Cardiovascular Study of Motion, University of West Attica, Athens, Greece; 5 Second Department of Neurology, School of Medicine, National and Kapodistrian University of Athens, Attikon University Hospital, Athens, Greece; 6 Deutsches Zentrum Für Neurodegenerative, Erkrankungen Rostock/Greifswald, Rostock, Germany; 7 Center for Transdisciplinary Neurosciences Rostock, Rostock University Medical Center, Rostock, Germany; 8 Translational Neurodegeneration Section Albrecht Kossel, Department of Neurology, Rostock University Medical Center, Rostock, Germany; 9 Department of Neurology, Rostock University Medical Center, Rostock, Germany

**Keywords:** ALS, Vagus nerve, Ultrasound, Autonomic dysfunction, Disease severity, Individual patient data meta-analysis

## Abstract

**Background:**

Several single-center studies proposed utility of vagus nerve (VN) ultrasound for detecting disease severity, autonomic dysfunction, and bulbar phenotype in amyotrophic lateral sclerosis (ALS). However, the resulting body of literature shows opposing results, leaving considerable uncertainty on the clinical benefits of VN ultrasound in ALS.

**Methods:**

Relevant studies were identified up to 04/2024 and individual patient data (IPD) obtained from the respective authors were pooled with a so far unpublished cohort (from Munich). An IPD meta-analysis of 109 patients with probable or definite ALS (El Escorial criteria) and available VN cross-sectional area (CSA) was performed, with age, sex, ALS Functional Rating Scale-revised (ALSFRS-R), disease duration, and bulbar phenotype as independent variables.

**Results:**

Mean age was 65 years (± 12) and 47% of patients (± 12) had bulbar ALS. Mean ALSFRS-R was 38 (± 7), and mean duration was 18 months (± 18). VN atrophy was highly prevalent [left: 67% (± 5), mean CSA 1.6mm^2^ (± 0.6); right: 78% (± 21), mean CSA 1.8 mm^2^ (± 0.7)]. VN CSA correlated with disease duration (mean slope: left − 0.01; right − 0.01), but not with ALSFRS-R (mean slope: left 0.004; mean slope: right − 0.002). Test accuracy for phenotyping bulbar vs. non-bulbar ALS was poor (summary receiver operating characteristic area under the curve: left 0.496; right 0.572).

**Conclusion:**

VN atrophy in ALS is highly prevalent and correlates with disease duration, but not with ALSFRS-R. VN CSA is insufficient to differentiate bulbar from non-bulbar ALS phenotypes. Further studies are warranted to analyze the link between VN atrophy, autonomic impairment, and survival in ALS.

**Supplementary Information:**

The online version contains supplementary material available at 10.1007/s00415-024-12601-z.

## Introduction

Amyotrophic lateral sclerosis (ALS) is a devastating neurodegenerative disease with a mean life expectancy of only 2 to 5 years after symptom onset [1]. While being conceptualized as an isolated pathology of the central nervous motor system for many years, there is now growing evidence supporting the hypothesis of ALS being a multisystem neurodegenerative disease. Neuropathological studies revealed multisystem neuronal degeneration via intraneuronal aggregation and axonal spreading of soluble forms of transactive response DNA binding protein 43 kDa (TDP-43) and, thus, shares some pathophysiological aspects with other neurodegenerative diseases, particularly frontotemporal dementia (FTD) [2, 3]. Usually TDP-43-proteinopathy first appears in the primary motor cortex before further spreading in a Prion-like propagating manner to various other and more distant regions, particularly lower motor neurons in the brainstem and the anterior horn of the spinal cord [3, 4]. Accordingly, besides progressive and irresistible upper and lower motor neuron loss, behavioral and cognitive symptoms as well as autonomic dysfunction with gastrointestinal, salivary and cardiovascular symptoms have recently been described as clinical manifestations of ALS [5–7]. The perception of ALS as a multisystem neurodegenerative disease is reinforced by recent studies showing that sensory abnormalities, as measured by skin innervation testing, parallel motor function decline and correlate with clinical ALS stages according to the King’s staging system [8].Yet, the current diagnostic frameworks including the revised El Escorial criteria, Awaji-Shima criteria and recent Gold Coast criteria, are still exclusively relying on clinical motor symptoms supported by classical needle electromyography with concomitant appearance of active denervation and reinnervation potentials [9–12].

Ultrasound measurement of vagus nerve (VN) cross-sectional area (CSA) was postulated to be a potential diagnostic biomarker for a primary bulbar manifestation of ALS in earlier studies (Tawfik et al., Holzapfel et. al.), while more recent work by Papadopoulou et. al. and Weise et. al. could not validate and replicate these findings [13–16]. However, Papadopoulou et. al. reported a correlation between the presence of VN atrophy and disease severity as well as disease duration, leading to the hypothesis that VN CSA might be a valuable and valid imaging biomarker for autonomic dysfunction in ALS. Interestingly, Dubbioso et al. recently showed a higher load of dysautonomia in bulbar ALS using electrophysiological studies and questionnaires, but not VN sonography [17]. Overall, there is, thus, opposing evidence on the diagnostic test accuracy of VN CSA for bulbar phenotype and its role as a surrogate of autonomic dysfunction in ALS. Given the considerable uncertainty on the practical benefits for the clinical use of VN ultrasound in ALS, we performed a single-center cases series and pooled individual patient data meta-analysis (IPD-MA) to clarify the diagnostic test accuracy and relevant confounders of VN CSA in ALS.

## Materials and methods

### Study design, search strategy, and study eligibility

In our study, we conducted an IPD-MA following the reporting guidelines of PRISMA-IPD [18]. We employed a comprehensive search strategy (Supplementary Material S1a) based on medical subject headings (MeSH) in the Medline, EMBASE, Clinical trials.gov, and Cochrane Library databases up to April 2024 to identify relevant studies involving probable or definite ALS patients with measurement of VN CSA at the thyroidal gland level. As the ultrasound imaging protocols for VN CSA varied, we only included studies with standardized measurements at the level of the thyroid gland, as this has been shown to have least statistical heterogeneity [19]. Consequently, studies with assessment at the carotid bifurcation or thyroid cartilage were excluded [19]. The review protocol was registered at PROSPERO (CRD42024532588).

### Data collection

We contacted the corresponding authors of eligible studies to request de-identified, individual participant data using a data extraction sheet. The requested variables were age, sex, bulbar phenotype, ALS Functional Rating Scale-revised (ALSFRS-R), disease duration, and bilateral VN CSA at the level of the thyroid gland [19].

For the Munich cohort, all consecutive cases between 09/2022 and 09/2023 with clinically probable or definite ALS with respect to El Escorial criteria were retrospectively screened for available VN measurement and relevant data were extracted from electronic health care records. At this center, high-resolution ultrasound was conducted using a 15-MHz transducer (L4-15, Esaote MyLab Omega, Genoa, Italy), adhering to protocols outlined in previously published literature [20]. To minimize inter-rater variability, a standard operating procedure detailing ultrasound settings and examination techniques has been established. Bilateral VN was scanned in the axial view and CSA was measured at the level of the thyroid gland (Supplementary Material S1b). Ethical approval for collecting data in the Munich cohort was obtained from the local ethics committee (protocol number 23–0833).

### Data pre-processing

The datasets were checked for consistency and were harmonized. Predictive mean matching was used as imputation method to account for missing data (20/1472 (1.4%) of overall data points).

### Risk of *bias* assessment

Using the Newcastle–Ottawa Scale (NOS), two independent reviewers (KJM and MLS) assessed the risk of bias of eligible studies. Disagreement was resolved by consensus. The NOS contains eight items and evaluates three dimensions (selection criteria, comparability, and outcome/exposure) with a maximum score of 9 [21]. Risk of bias was stratified into three categories (high, some concern, low), and visualized using the robvis tool [22].

### Statistical analysis and software

For continuous independent variables (age, sex, ALSFRS-R, disease duration), we used a two-staged method to accommodate potential non-linear associations with VN CSA as well as within-study correlation [23]. First, we modeled the relationship between one independent variable and VN CSA using fractional polynomials while adjusting for the remaining independent variables for each study center separately. Second, as a between-study analysis, random-effect models were used to pool estimates across centers. Finally, using Lowess plots, the predicted values of the respective independent variable were visualized as a function of VN CSA including 95% confidence intervals. Mean slopes were computed with numerical differentiation to express the correlation of independent variable and VN CSA numerically. With regards to the diagnostic accuracy for a bulbar ALS phenotype, we calculated descriptive statistics (true positives, true negatives, false positives, and false negatives) and computed sensitivity and specificity including 95% confidence intervals as measures of uncertainty using the Wilson method and a 0.5 continuity correction with the previously suggested threshold for VN CSA of 1.85 mm^2^ [14, 24]. Given the rather low number of studies available, an univariate approach [Proportional Hazards Model with Adjusted Profile Maximum Likelihood Estimator (APMLE)] was chosen for the meta-analysis and calculation of summary receiver operating characteristic (SROC) and its area under the curve (AUC) [25].

All analyses were performed using R (2023.06.1 + 524) with `mfp`, `randomForestSRC`, `missForest`, ‘mada’ and `mice` packages. ChatGPT (version 4) was used for error handling, repetitive programming, and overall optimization of code in R.

## Results

### Study population

**Fig. 1:**
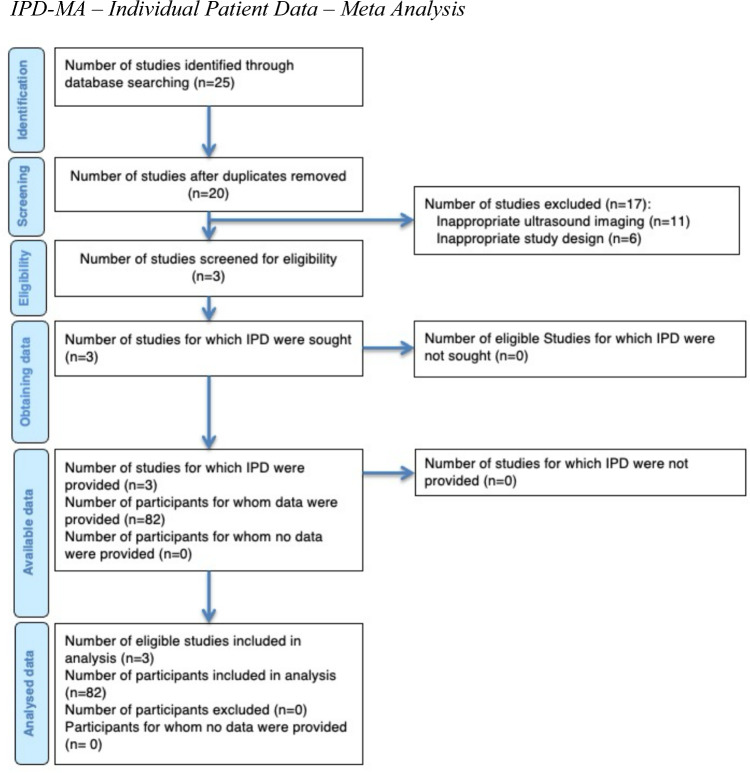
IPD-MA flow diagram. IPD-MA: individual patient data—meta analysis

Systematic review of existing literature revealed three eligible studies comprising a total of 82 individual patients (Fig. 1). Pooled with the unpublished case series from the Munich cohort, 109 datasets were available for final analysis. Baseline characteristics grouped by study center are depicted in Table 1. The mean age of participants was 65 years (± 12), and 45% were female. A bulbar phenotype was present in 47% (± 12) of all analyzed cases. The mean duration of symptoms was 18 months (± 18), with a mean ALSFRS-R score of 38 (± 7). The mean CSA of the VN was 1.8 mm^2^ (± 0.7) on the right and 1.6 mm^2^ (± 0.6) on the left side. Of all cases, VN atrophy was prevalent in 67% (± 5) on the left side and 78% (± 21) on the right side (Table 1).

**Table 1:** Baseline characteristics

Center	Munich *n* = 27	Athens *n* = 21	Leipzig *n* = 37	Augsburg *n* = 24	Total *n* = 109
Age (years), Mean (SD)	70 (11)	61 (11)	65 (12)	64 (10)	*65 (12)*
Female, Percent (SD)	67 (−)	24 (−)	46 (−)	33 (−)	*45 (19)*
Bulbar phenotype, Percent (SD)	33 (−)	62 (−)	51 (−)	42 (−)	*47 (12)*
Duration of disease (months), Mean (SD)	14 (8)	17 (17)	23 (23)	13 (10)	*18 (18)*
ALSFRS-R, Mean (SD)	38 (7)	35 (10)	38 (7)	42 (5)	*38 (7)*
Right VN CSA (mm^2^), Mean (SD)	1.9 (0.8)	1.7 (0.6)	1.7 (0.5)	1.9 (0.7)	*1.8 (0.7)*
Left VN CSA (mm^2^), Mean (SD)	1.9 (0.8)	1.5 (0.5)	1.5 (0.4)	1.8 (0.6)	*1.6 (0.6)*
Right VN atrophy, Percent (SD)	70 (−)	81 (−)	81 (−)	79 (−)	*78 (5)*
Left VN atrophy, Percent (SD)	37 (−)	86 (−)	73 (−)	71 (−)	*67 (21)*

Right vagus nerve atrophy < 2.39mm^2^, left vagus nerve atrophy < 1.87 mm.^2^(Abdelnaby R. et. al. 2022) [19]

*SD* standard deviation, *CSA* cross-sectional area

### Risk of *bias* assessment

Risk of bias was moderate to low for all three studies. Yet, the selection of the respective cohorts was not described in detail in any of the studies. (Fig. S1a in Supplementary Material S1).

### Diagnostic test accuracy for a bulbar phenotype

**Fig. 2:**
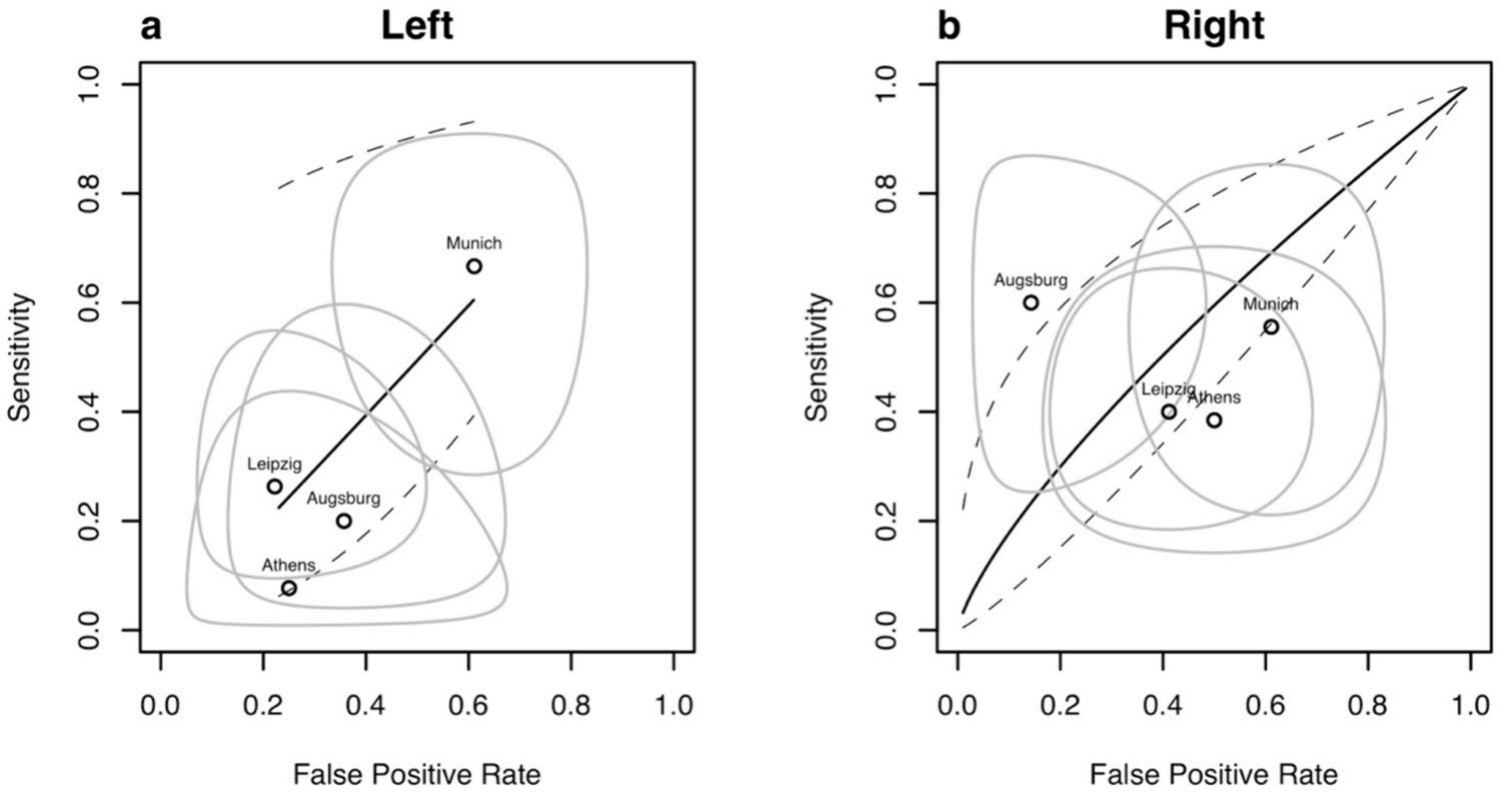
Diagnostic test accuracy of VN CSA for a bulbar phenotype of ALS. SROC (solid black line) with 95% confidence intervals (dotted line) for bulbar ALS derived from center-specific diagnostic test statistics [point estimates with 95% confidence intervals (black circles with surrounding grey elliptic lines)] of the left (**a**) and right (**b**) VN using a threshold of 1.85mm^2^ for CSA. *SROC* summary receiver operating curve, *ALS* amyotrophic lateral sclerosis, *CSA* cross-sectional area

At a threshold of 1.85mm^2^ for VN CSA, sensitivity and specificity exhibited great between-study heterogeneity and a high level of uncertainty (Fig. 2, Fig. S1d in Supplementary Material S1). Accordingly, meta-analysis with SROC revealed a low diagnostic value of VN CSA for bulbar ALS versus non-bulbar ALS (SROC AUC left VN: 0.496; SROC AUC right VN: 0.572) (Fig. 2).

### Confounders *of vagus* nerve CSA in ALS

**Fig. 3:**
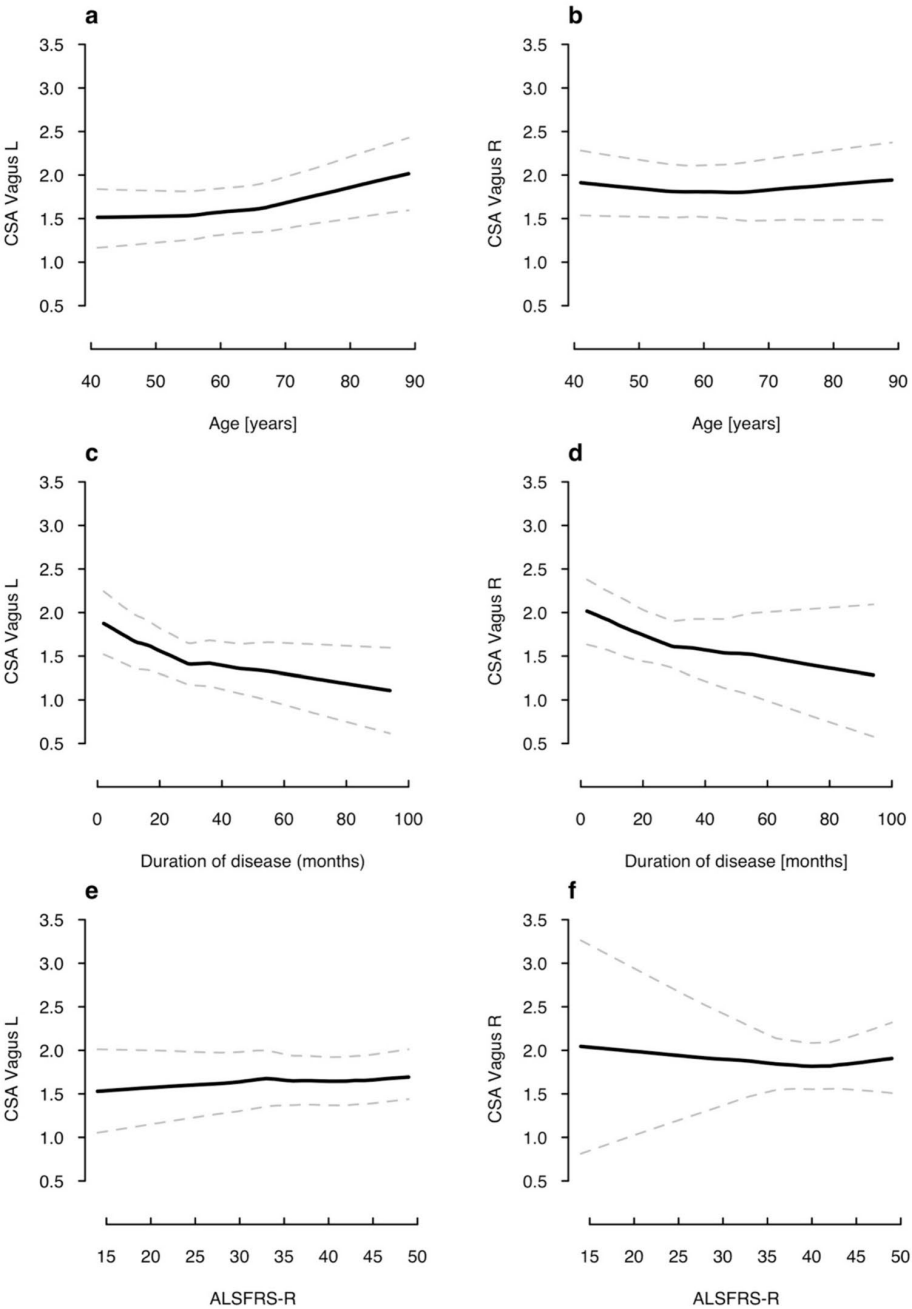
Random-effects model with 95% confidence interval illustrating correlation of VN CSA with age (**a**,** b**), disease duration (**c**,** d**), and disease severity (**e**,** f**). *CSA* cross-sectional area, *L* left, *R* right

To identify influencing factors other than a bulbar phenotype, we correlated the continuous variables age, disease duration, and ALSFRS-R with VN CSA. Interestingly, a positive correlation was found between left VN CSA and age (Fig. 3a: mean slope 0.01), whereas right VN CSA showed no relevant association with age (Fig. 3b: mean slope 0.0007). Bilaterally, a decrease in VN CSA was noted with longer disease duration (Fig. 3c, d: mean slope left − 0.01; mean slope right − 0.01). Notably, this was not paralleled by a strong correlation of VN CSA and ALSFRS-R on either side (Fig. 3e, f: mean slope left 0.004; mean slope right − 0.002).

## Discussion

In this IPD-MA, we present a thorough analysis of confounders of VN CSA and analyze its diagnostic test accuracy for a bulbar ALS phenotype. Our key findings can be summarized as follows: first, there is an overall high prevalence of bilateral VN atrophy in ALS. Second, VN CSA decreased markedly with disease duration, but did not show a clear association with functional decline as measured by overall ALSFRS-R. Third, while left VN CSA increased with age, we could not observe any such correlation between age and right VN CSA. Fourth, VN CSA exhibited no reliable diagnostic utility for the differentiation of bulbar versus non-bulbar phenotypes in ALS.

Given the high prevalence of VN atrophy in our IPD-MA, autonomic dysfunction in ALS involving the VN appears plausible. Previous studies suggest sudden cardiac death occurs in almost 10% of ALS cases, listing it as the third most frequent cause of death in ALS patients overall [26, 27]. A recent prospective longitudinal cohort study detected autonomic dysfunction in two-thirds of the ALS cohort at the time of diagnosis and an even higher prevalence of more than 80% in later stages of the disease [17]. Importantly, autonomic dysfunction also correlated with more rapid disease progression, later disease stages (measured via King´s clinical stages) and survival [17, 28]. Similarly, in a previous case–control series among ventilator-dependent ALS patients, a significant elevation of norepinephrine levels indicating continuous sympathetic hyperactivity as well as a high prevalence of circulatory collapse (35%) and sudden cardiac death (26%) was observed [29]. An earlier study, which performed cardiac [^123^I] MIBG scintigraphy in an ALS cohort, showed significantly higher washout ratios than in controls (median in ALS patients: 38.5, range 11.1–71.4 vs. in control patients: median 29.4, range 20.0–36.0) indicating chronic cardiac sympathetic hyperactivity, which was also associated with sudden cardiac death and reduced overall survival [30]. Yet, whether VN CSA is an adequate biomarker for autonomic instability in ALS remains questionable, as the direct correlation of VN CSA and the established biomarkers of autonomic dysfunction mentioned above has not been sufficiently investigated so far. As our data—with measurements at the thyroid level—provide insights into the VN segment below the motor branches, it can be assumed as best possible assessment of isolated autonomic nerve fiber quantity and should, thus, be implemented as a standard in further research on autonomic dysfunction in neurodegenerative diseases.

Disease duration correlated with VN CSA, whereas ALSFRS-R did not. This discrepancy may be explained by ALSFRS-R score’s focus on respiratory symptoms and limb motor function decline, while having a blind spot for non-motor functions, particularly autonomic functions, which VN CSA at the thyroid level explicitly measures. Also, ALSFRS-R as a surrogate of overall impairment might not be sensitive enough to detect VN-mediated dysfunction. Furthermore, while disease duration correlates with VN atrophy, our data does not provide insights into how disease progression (assessed via ALSFRS-R decline or estimates of the slope of decline) is correlated with VN atrophy. Currently, there is a lack of data in the literature regarding the onset of autonomic dysfunction and its association with clinical stages (such as the King’s staging system), which needs to be addressed in future studies [28]. Given the reported prevalence of cardiac events in later stages of ALS and the observed correlation between VN atrophy and disease duration, it is possible that autonomic symptoms were assessed too early in previous studies [26, 27]. Therefore, the reported prevalence of autonomic dysfunction in ALS might be underestimated. Alternative scales, such as the Scales for Outcomes in Parkinson’s Disease-Autonomic Dysfunction (SCOPA-AUT), the Non-Motor Symptoms Questionnaire (NMSQ) or the Composite Autonomic Symptom Score 31 (COMPASS 31) scale, may provide more comprehensive and standardized evaluation and should, thus, be performed longitudinally [31]. Using NMSQ to measure autonomic dysfunction in ALS clearly showed higher values in ALS patients compared to controls and it correlated with disease severity. However, overall NMSQ and the amount of named singular items were less than in Parkinson’s disease (PD) [32]. Also, a need for adaptation of these autonomic symptom scales for the use in ALS patients has been discussed [30]. Recently, a single-center case series with 40 ALS patients could not establish any association between VN CSA (at the thyroid cartilage level) and NMSQ, the autonomic sub-score of NMSQ or 1-year mortality [33]. However, in this, case-series NMSQ was only conducted once at the beginning of the study. In addition, only 1 out of 40 patients experienced cardiac death within 1 year of observation. Thus, the clinical implication of VN atrophy in ALS still remains poorly and insufficiently understood. In addition, VN atrophy has also been investigated in other neurodegenerative syndromes. A comparative meta-analysis with 11 studies and 409 patients with PD showed a significantly reduced VN CSA compared to controls (ranging between 0.67 to 2.75mm^2^ in PD patients) [34], with further studies also indicating a correlation of VN atrophy with parasympathetic dysfunction in PD [35]. Besides, robust electrophysiological evidence supports the role of the VN as a critical component in maintaining cardio-vagal balance in both animal models and human studies of chronic heart failure [36, 37]. Here, surrogates of autonomic parasympathetic dysfunction such as reduced HRV, which can also be observed in ALS patients, were modified using neuromodulation of either left or right VN [17, 36]. Preliminary results of studies investigating the effects of VN stimulation in PD showed improvement of gait and cognitive impairment; however, the effects on autonomic function in PD have not been clarified yet [38, 39].

Furthermore, we noted a smaller CSA of the left compared to the right VN, as reported in the literature for both the ALS and the non-ALS cohort [19, 33]. The difference in size has previously been attributed to asymmetric innervation. Both VNs contribute to cardiac innervation [37, 40, 41]. However, the right VN predominantly innervates the celiac plexus, anterior gastric plexus, colon, and small intestine, while the left VN terminates at the anterior gastric plexus [42]. Interestingly, we also observed a positive correlation of the left VN with age, although there are opposing results on that topic in the literature. In a previous aggregate meta-analysis on reference values for VN CSA in non-ALS patients, stratification by age was not possible [19]. Overall, in the absence of data on corresponding measures for autonomic dysfunction (such as specific questionnaires or instrumental assessments) and given that both sides show similar proportions of VN atrophy, comparable correlations with disease severity, and exhibit similar cardio-vagal effects, the question whether right or left VN makes the better biomarker for ALS, let alone autonomic imbalance in ALS, remains unclear.

It seems plausible that VN CSA at the thyroid level is not a valid diagnostic tool to differentiate bulbar from non-bulbar ALS phenotypes, since at that particular localization, VN is purely autonomic. VN motor fibers innervating laryngeal and pharyngeal muscles already leave the nerve one level above at the level of the thyroid cartilage [15, 16]. The previously reported association of VN CSA with bulbar ALS may have resulted from a selection bias toward a non-representative and unusual high rate of ALS patients with a primary bulbar phenotype (i.e., 47%) within a small overall cohort (*n* = 24). However, VN CSA measured at cartilage level did also not discriminate ALS patients with present bulbar symptoms (*n* = 21) from those without bulbar symptoms (*n* = 19), even though the VN CSA at this location includes the motor (recurrent laryngeal) fibers [30]. On the other hand, phrenic nerve CSA were clearly smaller in ALS patients with bulbar phenotype [30]. Still, further longitudinal prospective studies with increased sample size are needed to draw definite conclusions on that particular topic.

Despite the insights gained from our study, several limitations should be acknowledged. The still relatively small study size, although bolstered by available patient-level data, may limit the generalizability of our findings. In addition, heterogeneity was high among the participating centers and given the limited description of cohort selection of the included studies, selection bias might be of particular concern. Moreover, we had to exclude two potential studies on VN ultrasound in ALS, as they measured at different anatomical landmarks (i.e., thyroid cartilage and carotid bulbus) [30, 31] given recent reports suggesting significant differences in CSA at those levels compared to the thyroid level in non-ALS patients [19]. Due to the retrospective nature of this study, the incomplete data on both patients’ longitudinal trajectories, and missing corresponding measures of autonomic dysfunction, we cannot draw any conclusions on the association of VN atrophy with disease progression (i.e., ALSFRS-R decline) and/or autonomic symptoms of ALS patients.

## Conclusion

The high prevalence of VN atrophy in ALS and its association with disease duration might indicate a role of VN CSA as a non-invasive, broadly available quantitative measure of autonomic instability in ALS. When measured at the thyroid level, diagnostic test accuracy for a bulbar versus non-bulbar phenotype seems insufficient. Future prospective multi-center studies with standardized ultrasound protocols are needed to elucidate the role of VN atrophy as a diagnostic and prognostic biomarker for autonomic impairment and survival in ALS.

## Supplementary Information

Below is the link to the electronic supplementary material.

Supplementary file1 (DOCX 4234 KB)

## Data Availability

Anonymized data from this study are available from the corresponding author on reasonable request.
