# Supplementary material for: Diagnostic accuracy and confounders of vagus nerve ultrasound in amyotrophic lateral sclerosis—a single-center case series and pooled individual patient data meta-analysis
Source: J Neurol. 2024 Jul 31;271(9):6255–63. doi: 10.1007/s00415-024-12601-z (PMC11377580; doi:10.1007/s00415-024-12601-z)
Supplement: Supplementary file 1 — Supplementary file1 (DOCX 4234 KB) [file 415_2024_12601_MOESM1_ESM.docx]

**Diagnostic accuracy and confounders of vagus nerve ultrasound in amyotrophic lateral sclerosis – a single-centre case series and pooled individual patient data meta-analysis**

Katharina J. Müller^1*^, Moritz L. Schmidbauer^1*^, Sonja Schönecker^1^, Katharina Kamm^1^, Johann O. Pelz^2^, Korbinian Holzapfel^3^, Marianna Papadopoulou^4^, Eleni Bakola^4^, Georgios Tsivgoulis^5^, Markus Naumann^3^, Andreas Hermann^6,7,8^, Uwe Walter^6,7,9^, Konstantinos Dimitriadis^1^, Peter Reilich^1^, Florian Schöberl^1^

**Contributed equally.*

Affiliations

^1^ Department of Neurology with Friedrich Baur Institute, LMU University Hospital, LMU Munich, Germany

^2^ Department of Neurology, Leipzig University Hospital, Leipzig, Germany

^3^ Department of Neurology and Clinical Neurophysiology, University of Augsburg, Augsburg, Germany

^4^ Department of Physiotherapy, Laboratory of Neuromuscular and Cardiovascular Study of Motion, University of West Attica, Athens, Greece

^5^ Second Department of Neurology, National and Kapodistrian University of Athens, School of Medicine, Attikon University Hospital, Athens, Greece

^6^ Deutsches Zentrum für Neurodegenerative Erkrankungen Rostock/Greifswald, Rostock, Germany

^7^ Center for Transdisciplinary Neurosciences Rostock, Rostock University Medical Center, Rostock, Germany

^8^ Translational Neurodegeneration Section "Albrecht Kossel," Department of Neurology, Rostock University Medical Center, Rostock, Germany

^9^ Department of Neurology, Rostock University Medical Center, Rostock, Germany

Correspondence
Florian Schöberl, Department of Neurology, University Hospital LMU Munich
Tel +49-4400-0, [florian.schoeberl@med.uni-muenchen.de](mailto:florian.schoeberl@med.uni-muenchen.de)

Keywords
ALS, vagus nerve, ultrasound, autonomic dysfunction, disease severity, individual patient data meta-analysis

**Search terms**

Pubmed: ((Amyotrophic lateral sclerosis) AND (ultrasound)) AND (vagus nerve)

Embase: (amyotrophic lateral sclerosis and ultrasound and vagus nerve).af.

Cochrane: (als and ultrasound and vagus nerve)

Clinical trials.gov: Condition: Amyotrophic lateral sclerosis, Intervention: Ultrasound


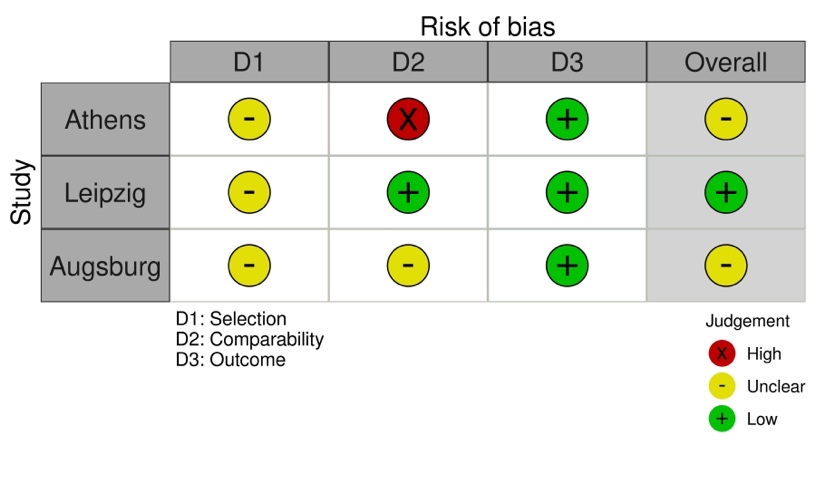
**Fig.** **S1a** Risk of bias of eligible studies

Risk of bias for selection (D1), comparability (D2) and exposure (D3). High risk of bias (red): D1 0-1/4 points, D2 0/2 points, D3 0-1/3 points; some concern for risk of bias (yellow): D1 2-3/4 points, D2 1/2 points D3 2/3 points; low risk of bias (green): D1 4/4 points, D2 2/2 points, D3 3/3 points

**b**

**a**

**Fig. S1b** Representative sonographic imaging of VN CSA at the level of the thyroid gland

Depiction of right VN (marked with a white arrowhead) with 4.0mm^2^ CSA (***a***) and 1.5mm^2^ (***b***) respectively.

*VN-vagus nerve; CSA-cross sectional area; CCA-common carotid artery; IJV-internal jugular vein; TG-thyroid gland; SCM-sternocleidomastoid muscle.*

**a**

**b**

**c**

**d**

**e**

**f**

**Fig. S1c** Correlation of VN CSA with duration of disease (***a,b***), ALSFRS-R (***c,d***) and age (***e,f)*** for each study

**a**

**b**

**c**

**d**

**Left**

**Right**

**Left**

**Right**

**Fig. S1d** Sensitivity and specificity for bulbar phenotype at 1.85mm^2^ CSA of VN
